# Sensitive protein alignments at tree-of-life scale using DIAMOND

**DOI:** 10.1038/s41592-021-01101-x

**Published:** 2021-04-07

**Authors:** Benjamin Buchfink, Klaus Reuter, Hajk-Georg Drost

**Affiliations:** 1 Computational Biology Group, Max Planck Institute for Developmental Biology, Tübingen, Germany; 2 Max Planck Computing and Data Facility, Garching, Germany

**Keywords:** Genomic analysis, Sequencing, Software, Computational biology and bioinformatics, Genome informatics

## Abstract

We are at the beginning of a genomic revolution in which all known species are planned to be sequenced. Accessing such data for comparative analyses is crucial in this new age of data-driven biology. Here, we introduce an improved version of DIAMOND that greatly exceeds previous search performances and harnesses supercomputing to perform tree-of-life scale protein alignments in hours, while matching the sensitivity of the gold standard BLASTP.

## Abstract (web summary)

An updated version of DIAMOND uses improved algorithmic procedures and a customized high-performance computing framework to make seemingly prohibitive large-scale protein sequence alignments feasible.

## Main

Within the next decade, The Earth BioGenome Project^1,2^ aims to sequence and assemble the reference genomes for ~1.5 million of the 10–15 million known eukaryotic species that inhabit our planet. Current sequence search algorithms and software tools would be impractical for analyzing data of this magnitude when aiming to retain sensitivity similar to the gold standard BLASTP^3^. In an experimental study we estimated that querying the National Center for Biotechnology information (NCBI) non-redundant (nr) database (~280 million sequences) against the UniRef50 database (~40 million sequences) using BLASTP would require more than 2 months even on a supercomputer equipped with 20,800 cores (Methods). However, the newly developed version of DIAMOND can now accomplish the same task in several hours, with an alignment sensitivity that matches BLAST. We overcome this computational bottleneck and enable sensitive large-scale protein searches on a tree-of-life scale by introducing improved algorithmic procedures and a customized high-performance computing framework, which incorporate optimized distributed computing, double indexing and multiple spaced seeding. This version of DIAMOND is available as Open Source Software under the GPL3 license (http://www.diamondsearch.org).

DIAMOND is a fast and sensitive protein aligner that was initially developed for metagenomics applications to achieve ultra-fast alignments at the cost of alignment sensitivity, compared with the gold standard, BLAST. Although DIAMOND is proven to be practical for many metagenomics studies that also often rely on *k*-mer information for annotation and classification^4^, most functional and phylogenomic studies rely heavily on high alignment sensitivity to obtain useful insights about the functional conservation of proteins and their evolutionary divergence along phylogenetic lineages. For data-intensive studies in these fields, BLAST remains the tool of choice due to its paramount alignment sensitivity.

Here, we introduce a greatly improved version of DIAMOND that provides two sensitivity modes, --very-sensitive and --ultra-sensitive, which will enable data-intensive comparative genomics research such as tree-of-life scale tracing of protein evolution^5^, gene age inference^6,7^, and functional annotation of genes and gene families^8^ to be carried out with the same accuracy as BLAST, but with an 80–360-fold computational speedup. In --ultra-sensitive mode, DIAMOND (v2.0.7) achieves this BLAST-like sensitivity milestone while reducing the computational run time of BLASTP-heavy studies from months to hours.

This version of DIAMOND is different from other protein aligners and its older versions in that it focuses on ultra-fast but sensitive protein searches that can scale with sequencing efforts; for example, to meet the demands of the large-scale Earth BioGenome Project and analogous bulk sequencing projects. Alternative tools such as BLASTP (ref. ^3^), USearch (ref. ^9^), LAST (ref. ^10^) or MMSeqs2 (ref. ^11^) are also optimized to run fast protein alignments, but still require longer computation times and, with the exception of BLAST, are less sensitive than DIAMOND when dealing with very large datasets. These tools already experience limitations when they try to handle searches at the scale of the NCBI nr database, which currently contains the largest collection of sequences, representing genomic information for ~12,000 eukaryotic species. Therefore we sought to build a protein search infrastructure that can accommodate the demands of sensitive homology searches on this exponentially growing dataset of sequenced species.

DIAMOND now achieves this goal by providing four different levels of alignment sensitivity and by optimizing two distinct computational paradigms. First, it leverages an ultra-fast integration of algorithmic steps optimized for the latest generation of computer architectures that are designed to function optimally when dealing with massive query and subject databases. Second, it harnesses high-performance computing (HPC) and cloud computing by providing a powerful distributed computing implementation customized for large-scale protein searches, incorporating our new DIAMOND search scheme (Methods). In summary, our method is based upon on-the-fly double indexing (in which both the reference database and the query are indexed for comparison) and hash join on the seed space spanned by up to 64 multiple spaced seeds (seeds that are extracted from the sequence according to a pattern of ‘match’ and ‘don’t care’ positions) to greatly improve the specificity of seeding relative to a baseline strategy. Furthermore, double indexing focuses the comparison operations with respect to a seed and enables the operations to be streamed through the CPU in an efficient, cache-aware manner, avoiding the memory latency bottleneck of a classical single-indexed seed lookup approach. A chain of heuristic filter stages that makes heavy use of vector instructions is designed to gradually eliminate spurious hits, while passing on potentially significant alignments to a vectorized Smith–Waterman extension.

**Fig. 1:**
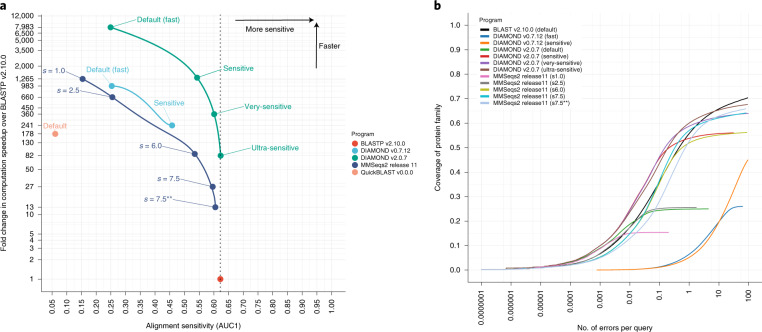
Benchmark of DIAMOND, MMSeqs2 and BLASTP using various sensitivity modes. Computational speedup and alignment sensitivity comparisons were carried out between the new version of DIAMOND, v2.0.7 (using default, --sensitive, --very-sensitive and --ultra-sensitive modes), the old version of DIAMOND, v0.7.12 (using default and --sensitive modes), MMSeqs2 release 11 (using modes *s* = 1.0, *s* = 2.5, *s* = 6.0, *s* = 7.5, *s* = 7.5** with --max-seqs 100000), BLASTP v2.10.0 and QuickBLAST v0.0.0. **a**, Alignment sensitivity (AUC1) measured as the fraction of the query’s protein family covered until the first false positive, averaged over all queries in the benchmark dataset. Dashed vertical line, alignment sensitivity level of BLASTP v2.10.0 (AUC1 = 0.622). **b**, ROC curves of the same benchmark showing the true average error rate per query versus the average coverage of the protein family, depending on the *e*-value threshold. Source data

We demonstrate the search capabilities of DIAMOND (v2.0.7) by systematically comparing its performance against BLASTP (v2.10.0) and MMSeqs2 (release 11), and against an older version of DIAMOND (v0.7.12), all of which are currently the most promising alternatives for sensitive tree-of-life scale protein searches (Fig. 1). To create a benchmark dataset covering annotated protein sequences spanning the full diversity of the tree of life, we downloaded the NCBI nr database (25 October 2019) and annotated each protein sequence in accordance with their SCOPe (structural classification of proteins–extended) domains^12^ (http://scop.berkeley.edu/) (Methods). Establishing a ground truth on the basis of SCOP domains has been considered the gold standard for benchmarking protein aligners^13^. As a result of the annotation, we obtained a query dataset of ~1.7 million protein sequences covering ~1,000 representative sequences for each SCOPe superfamily. Furthermore, we annotated the UniRef50 database^14^ (accessed 14 September 2019) following the same procedure to serve as a reference database for the benchmark.

It is important to note that some previous performance benchmarks between older versions of DIAMOND and other aligners^15^ used small benchmark datasets for the comparison with DIAMOND. As stated earlier, DIAMOND is optimized for searches using large query and reference databases. Therefore valuable benchmarking insights can only be achieved when comparing DIAMOND and other tools using large benchmark datasets, rather than focusing on small query or reference examples.

We ran DIAMOND (v2.0.7) in all four sensitivity modes using our SCOPe-annotated benchmark dataset as a query against the UniRef50 database, and compared its computational performance and level of sensitivity against analogous runs performed with BLASTP (v2.10.0), MMSeqs2 (release 11) and DIAMOND (v0.7.12). Figure 1 shows the benchmarking results of these alignments against the UniRef50 database. For each tool, we show the performance increase of the respective search algorithm over BLASTP against the average recall of a query’s protein family until the first false positive (Fig. 1a), and the corresponding receiver operating characteristic (ROC) curve (Fig. 1b). We found that DIAMOND (v2.0.7) computed alignments 12–15-fold faster than MMSeqs2 (release 11) while maintaining similar sensitivity levels. When the new DIAMOND was compared with older versions of DIAMOND^16^ (v0.7.12) we observed a 6–8-fold speedup, while the old DIAMOND was also far behind the other benchmarked tools in terms of sensitivity. When comparing DIAMOND (v2.0.7) to BLASTP (v2.10.0) we observed an ~8,000-fold speedup when using the least sensitive mode, and still an 80-fold speedup when running DIAMOND (v2.0.7) with a sensitivity level matching that of BLASTP (ultra-sensitive mode). Closer inspection of the trade-off between sensitivity and specificity on the basis of ROC curves (Fig. 1b) shows that DIAMOND (v2.0.7) in both the very-sensitive and ultra-sensitive modes maintained equal or marginally better sensitivity than BLAST at low error rates, while being only slightly surpassed by BLAST at error rates of >1 false positive per query (in which searches at error rates of >1 have only rare practical applications). We also conclude that the more sophisticated repeat masking used by DIAMOND (v2.0.7) (Methods) enables lower true error rates at similar sensitivity levels.

In addition, we compared older versions of BLASTP (v2.2.31; 2015) to the 2019 version of BLASTP (v2.10.0) and found that the 2019 version of BLASTP was fourfold faster than its 2015 version. Although this speedup is impressive, we are not able to envision a scenario in which this rate of increase will enable tree-of-life scale protein alignments when dealing with sequences from millions of eukaryotic species.

**Fig. 2:**
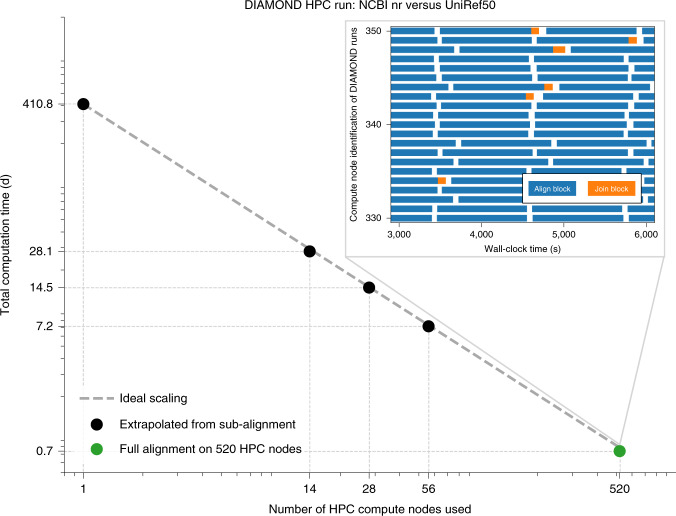
Strong scaling of DIAMOND on up to 520 nodes (20,800 cores) of the Cobra supercomputer of the Max Planck Society. The parallel scalability of DIAMOND in a distributed computing environment (supercomputer) is shown. Runs are performed in --ultra-sensitive mode, using the NCBI nr database (~281 million sequences) as the query database and the UniRef50 database (~39 million sequences) as the reference database. The full alignment was performed on 520 nodes (20,800 cores total), finishing in less than 18 hours of wall-clock time. On 1, 14, 28 and 56 nodes, only a subset of the query blocks could be processed, and the time for the full alignment was linearly extrapolated for each node count. An almost ideal scaling is observed, with a small super-linear effect caused by the caches of the parallel input–output system. To illustrate the massive parallelism, the inset shows a zoomed-in view of the sequence of tasks that DIAMOND (v2.0.0) has performed over time on 20 of the 520 nodes, indicated by blue rectangles (alignment process) and orange rectangles (join operations). White spaces encode the input–output activity on the supercomputer’s shared parallel file system (Extended Data Fig. 1). Source data

**Extended Data Fig. 1:**
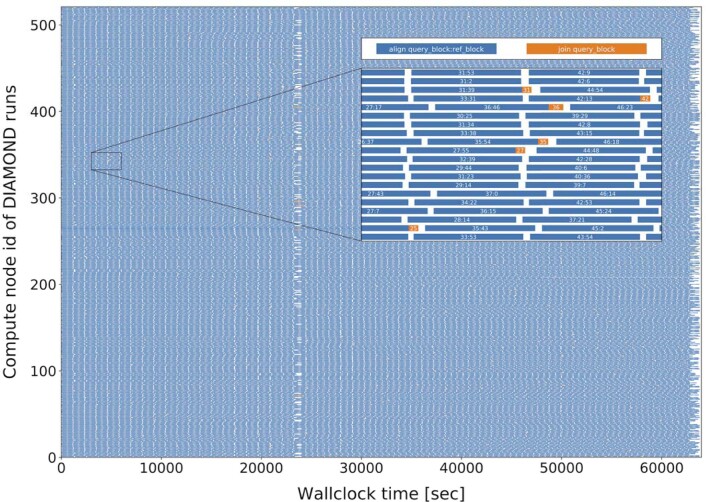
Detailed visual trace of a massively parallel DIAMOND run on 520 nodes (41,600 threads) of the Cobra supercomputer of the Max Planck Society. To illustrate the scalability of DIAMOND (v2.0.0) in a distributed computing environment (supercomputer), line-by-line worker tasks are shown individually for each worker node (the detailed version of Fig. 2). The sequence of tasks DIAMOND (v2.0.0) has performed over time is indicated by blue rectangles and orange rectangles, in which blue rectangles denote the alignment process and orange rectangles represent join operations. The numbers within the rectangle indicate the indices of the individual query chunks and of their respective reference chunks. White spaces encode the IO activity on the supercomputer’s shared parallel file system. The run shown here was performed in–ultra-sensitive mode and used the full NCBI non-redundant database as the query database, and the UniRef50 database as the reference database, finishing in below 18 hours of wallclock time. The result demonstrates DIAMOND’s efficient distribution of massively parallel work packages at scale, showing that all workers finish around the same time without creating a significant load imbalance (Experimental Study).

To demonstrate the capabilities of our tool when supported by an HPC infrastructure, we aligned all 281 million protein sequences from the NCBI nr database against the UniRef50 database, which consists of 39 million sequences, using DIAMOND (v2.0.7) in ultra-sensitive mode on the Cobra supercomputer of the Max Planck Society. This comprehensive comparison across all domains of life was computed in less than 18 hours using 520 compute nodes (Fig. 2 and Extended Data Fig. 1), compared with an estimated 2 months with BLAST.

**Extended Data Fig. 2:**
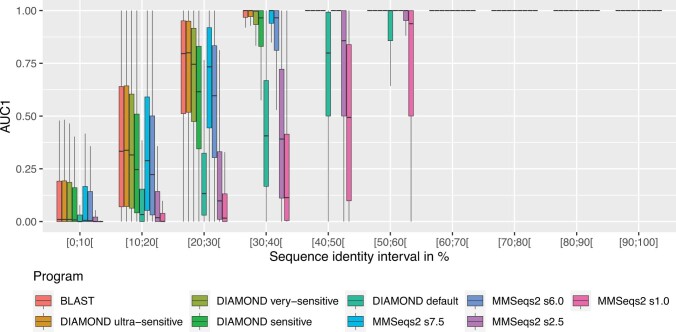
Assessment of true positive alignments. AUC1 sensitivity as reported for our main benchmark, resolved by sequence identity of the query-subject association under our SCOPe annotation (middle = median, hinges=25%/75% quantiles, lower/upper whisker = smallest/largest observation greater/less than or equal to lower/upper hinge -/+ 1.5 * IQR). Source data

**Extended Data Fig. 3:**
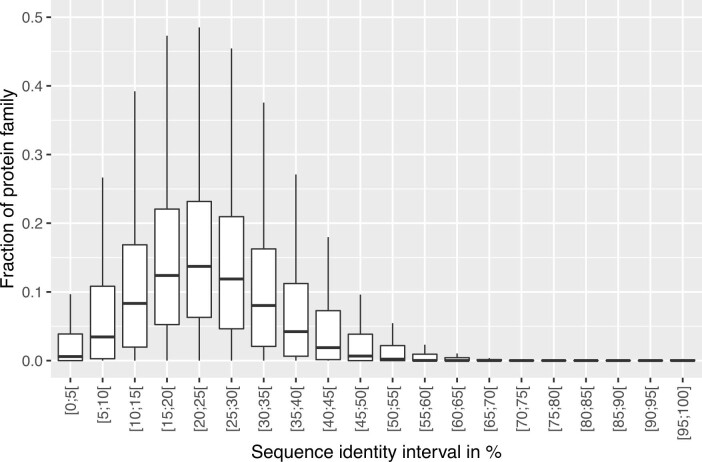
Assessment of protein family associations. The distribution (shown in 5% bins) of a query’s protein family member associations with respect to the sequence identity of the corresponding Needleman Wunsch alignments between the annotated ranges (middle = median, hinges=25%/75% quantiles, lower/upper whisker = smallest/largest observation greater/less than or equal to lower/upper hinge −/+ 1.5 * IQR). Source data

**Extended Data Fig. 4:**
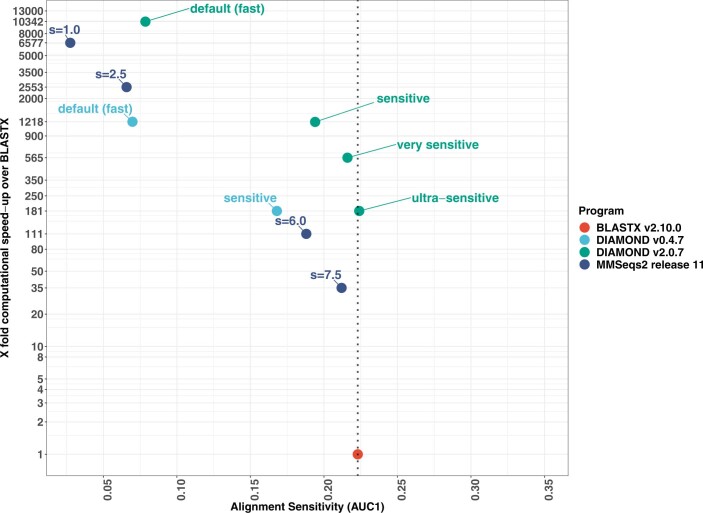
Metagenomic benchmark results using Illumina HiSeq 4000 paired end sequencing (2x150 bp) reads from Stewart et al., 2019. Computational speedup and alignment sensitivity comparisons for translated searches of 150bp Illumina short reads from rumen metagenome samples (Supplementary Benchmark 1). Alignment sensitivity (AUC1) is measured as the fraction of the query’s protein family covered until the first false positive, averaged over all queries in the benchmark dataset. The y-axis denotes the x-fold computational speedup achieved over BLASTX v2.10.0. Source data

**Extended Data Fig. 5:**
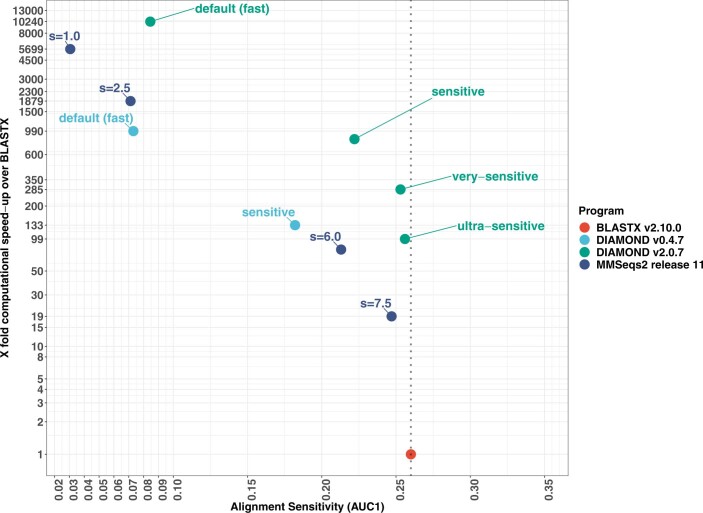
Metagenomic benchmark results using Illumina HiSeq 2500 paired end sequencing (2x250 bp) reads from Bahram et al., 2018. Computational speedup and alignment sensitivity comparisons for translated searches of 250bp Illumina short reads from topsoil metagenome samples (Supplementary Benchmark 2). Alignment sensitivity (AUC1) is measured as the fraction of the query’s protein family covered until the first false positive, averaged over all queries in the benchmark dataset. The y-axis denotes the x-fold computational speedup achieved over BLASTX v2.10.0. Source data

**Extended Data Fig. 6:**
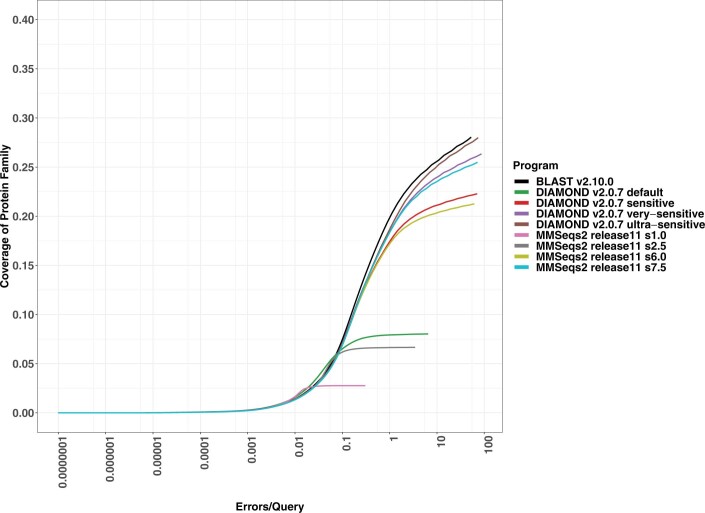
ROC curves for metagenomic benchmark using Illumina HiSeq 4000 paired end sequencing (2x150bp) reads from Stewart et al., 2019. We show the true average error rate per query (x-axis) against the average coverage of the protein family (y-axis) depending on the e-value threshold for Supplementary Benchmark 1. Source data

**Extended Data Fig. 7:**
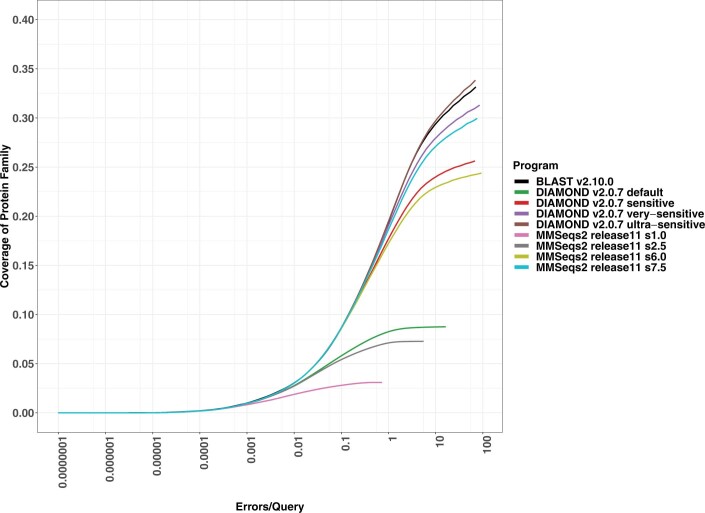
ROC curves for metagenomic benchmark using Illumina HiSeq 2500 paired end sequencing (2x250bp) reads from Bahram et al., 2018. We show the true average error rate per query (x-axis) against the average coverage of the protein family (y-axis) depending on the e-value threshold for Supplementary Benchmark 2. Source data

For further evaluation, we report the alignment sensitivity resolved by sequence identity (Extended Data Fig. 2), the distribution of homologs across identity bins (Extended Data Fig. 3), and the results of two supplementary benchmarks based on short reads (Extended Data Figs. 4–7).

Here, we introduce a comprehensive sequence search framework based on an extensively improved version of DIAMOND (v2.0.7) that enables users to handle the accelerating growth of sequence information for data-driven comparative and functional genomics studies. We designed this framework to meet the computational demands of future high-sensitivity sequence searches, to gain fundamental insights into protein evolution and molecular phylogenetics.

## Methods

### Algorithmic overview of DIAMOND

#### Double indexing

DIAMOND uses the double-indexing approach, in conjunction with multiple spaced seeds^17^, to optimize the handling of large query and large reference databases. In the first step, tables of seed–location pairs are built for query and reference sequences. Next, matching seeds are computed using a hash join technique that conducts recursive radix clustering of both tables until a hash table for the query data fits into the cache, at which point the rest of the join is computed by hashing^18^. We found this approach to be faster than the sorting method used by older versions of DIAMOND^16^, especially given that a full sorting of the reference table is avoided for smaller query datasets.

The double-indexing algorithm is designed to be cache aware, given that the data associated with one seed need to be loaded for comparison from memory only once, while the classical index-based linear seed lookup suffers from poor data locality. Additionally, our on-the-fly indexing method enables efficient use of multiple spaced seeds by processing the shapes one at a time and not requiring the index tables for all shapes to be present in memory simultaneously, while also avoiding expensive seed lookups through our cache-friendly hash join implementation.

DIAMOND (v2.0.7) uses two seed shapes of weight 10 for its fast mode, 16 shapes of weight 8 and 14 shapes of weight 7 for its sensitive and very-sensitive modes, respectively, and 64 shapes of weight 7 for its ultra-sensitive mode. The seed shapes were computed using SpEED^19^. Even when operating with 64 shapes, the run time generation of the indices, together with the join computation, take up less than 1% of the total run time of the program. When processing the NCBI nr database, the total size of these indices would be 123 billion letters × 9 bytes per entry × 64 shapes, which is ~64 TB if kept in memory or written to disk, while DIAMOND (v2.0.7) requires less than 16 GB of RAM when running in ultra-sensitive mode. This shows that DIAMOND does not require expensive computing infrastructures and can be operated with modest hardware resources if needed. Because of the runtime indexing, DIAMOND maintains disk-based database files that contain only the reference sequences, and can optionally also use BLAST databases (since v2.0.8).

#### Hamming distance filter

In the first stage of the sequence comparison process, a hamming distance computation between a query sequence and a subject sequence is performed at all seed hit locations in a 48-letter window encompassing the hit. We optimized this procedure using a chain of SSE (streaming single-instruction multiple-data (SIMD) extensions) pcmpeqb, pmovmskb and popcnt instructions to achieve a tenfold decrease in computation time compared with an ungapped alignment incorporating a scoring matrix, while reducing the number of hits by 1–2 orders of magnitude. A sensitivity-level-dependent cut-off for the hamming distance that can also be manually set by the user determines whether a hit is passed to the next filter stage.

We further extend our initial approach, introduced in the original version of DIAMOND^16^, and maximize the filtering throughput by using a loop-tiling strategy to incorporate the cache hierarchy and address the fact that the data associated with a single seed may exceed the cache capacity in the new very-sensitive and ultra-sensitive modes of DIAMOND (v2.0.7). We also load the 48-letter windows at the query and subject locations into linear buffers prior to running the all-versus-all hamming distance computation, to make best use of the hardware prefetcher and to avoid any random memory access.

#### Ungapped extension

After the hamming distance stage, the next step in the pipeline computes ungapped extensions at the seed hit locations. This procedure is vectorized using AVX2 instructions, aligning one query against up to 32 subject sequences. After 32 subject sequences are loaded into AVX2 registers, a 32 × 32 byte matrix transposition is computed using a series of 160 unpack instructions, such that 32 letters of different subjects are interleaved into one SIMD register, and the match scores can be loaded along the query. A sensitivity-level-dependent *e*-value threshold determines the hits that will be passed to the next stage.

#### Leftmost seed filter

Due to its double-indexing algorithm, DIAMOND may find the same alignment multiple times independently during the search stage. These redundant hits need to be filtered out to avoid an excessive use of temporary disk space. DIAMOND accomplishes this task by inspecting the local ungapped alignment for seed hits to the left of the hit that is currently being processed, as well as seed hits by previously processed shapes. If such a hit is found, DIAMOND notices the repetition and the current hit is discarded. Given that this procedure entails checking against up to 64 different seed shapes, we further optimized this process by incorporating a precomputed lookup table that stores information on whether any of the processed shapes will hit a given bit-encoded match or mismatch pattern, thus enabling the same check to be performed in one pass over the local hit pattern.

#### Adaptive ranking

Given that the typical application of an aligner will require the reporting of a certain number of best alignments (hits) for each query (as set on the command line using the --max-target-seqs option), DIAMOND makes use of this parameter to control the computational effort spent on seed extension and avoid having to compute gapped extensions for all seed hits. To this end, after the seed search within target sequences has been concluded, we determine a tentative order of target hits with respect to a single query. In the present case, this ranking procedure uses the ungapped extension scores at seed hits to assign a linear order to the targets. DIAMOND sorts the target list by ungapped extension score (from best to worst) for each target, similar to the way in which MMSeqs2 uses its ungapped extension-derived prefilter scores. Although MMSeqs2 will then compute Smith–Waterman extensions for a fixed number of best targets (as set using the --max-seqs parameter), DIAMOND uses a dynamic criterion to halt evaluation of further targets. We refer to this dynamic approach as adaptive ranking, which improves the DIAMOND reporting accuracy compared with the static criterion used by MMSeqs2, while providing a less biased and more data-adapted filtering procedure. The ranked list is processed in chunks of 400 targets (configurable on the command line using ext-chunk-size), for which extensions are computed. If no extension in the current chunk yields a significant alignment under the user-specified reporting criteria, computation of further extensions for the query is aborted, otherwise the next chunk of targets will be processed.

#### Gapped extension filter

Given that computing full Smith–Waterman^20^ extensions is expensive, we have developed a fast heuristic algorithm designed to estimate a gapped alignment score and discard hits that most probably do not meet the user-set reporting threshold. We use a query profile data structure in the same way as the vectorized Smith–Waterman algorithm introduced by Farrar^21^, which is an array for each of the amino acid letters that stores the scores along the query against the given residue. We then use AVX2 instructions to sum up these scores along diagonals of the dynamic programming matrix, thus computing local ungapped extension scores on diagonals. This approach ignores gaps in the alignment and therefore eliminates intra-register data dependencies. With its minimal logic, our heuristic achieves a throughput ~fivefold faster than a Smith–Waterman computation using the vectorized SWIPE method^22^. Nevertheless, ungapped scores on the diagonals can be used to estimate a gapped extension score by thresholding and computing a one-dimensional dynamic program that disregards the location of the diagonal segments. Although this simplifying assumption leads to an overestimation of the true alignment score most of the time, the heuristic is still able to reduce the number of spurious hits by one order of magnitude in the most sensitive alignment mode. If required by the user, this filter step can be disabled using the option gapped-filter-evalue 0.

#### Chaining

Chaining is the computation of a dynamic program at the level of diagonal segments instead of at the base or residue level, and has been used successfully in DNA alignment tools such as minimap2 (ref. ^23^). DIAMOND (v2.0.7) introduces the use of chaining on protein sequences. The result of the chaining computation is used to infer a scaffold for the optimal alignment and to determine the band geometry for a banded Smith–Waterman algorithm^20^.

Chaining can be simplified on DNA sequences by considering only diagonal segments of exact matches. However, this is not possible for protein sequences, which makes this computation substantially more elaborate. DIAMOND solves this problem by sorting the diagonal segments obtained by the ungapped extension stage on the starting position in the subject, and constructs a graph in which nodes represent diagonal segments and edges denote diagonal shifts (gaps) by computing pairwise connections between the diagonal segments in one left-to-right pass. Such pairwise connections are then stored as graph edges, incorporating their inbound and outbound coordinates to prevent invalid chains and to allow zigzag connections in which the optimal path repeatedly shifts between the same two diagonal nodes. A red–black tree for the nodes ordered on the diagonal is used to quickly access the most proximal nodes and candidates for determining a connection. For each node, the best score of a local alignment ending in that node is stored, the maximum of which yields the final score estimate and end point for backtracing of the approximate optimal alignment.

#### Banded SWIPE

The final extensions are computed using a modified version of the vectorized SWIPE (ref. ^22^) approach that accommodates banding. Due to their design, both the SWIPE and the ‘striped’ SIMD vectorization^21^ algorithms do not easily allow banded alignment, resulting in the need for an O(*n*²) computation in proportion to the length of the query and subject sequences. We vectorize the alignment of a query against up to 32 subjects by overlaying the banded dynamic programming matrix columns of the subjects based on their query ranges (the query coordinate interval [*i*_0_,*i*_1_] that corresponds to a slice of the given column with the subject’s band). Given that the bands of the subjects are different, this cannot be fitted perfectly into the register, but reaches a register load efficiency of 80–90% for larger databases. All extensions are computed using 8-bit scores and are repeated when an overflow is detected, unless an alignment score of >255 is already known from previous stages.

Alignments are scored using the BLOSUM62 matrix by default. In addition, we also use a method of composition-based score adjustments^15^ that is designed to increase the specificity of the scoring procedure. If required, DIAMOND (since v2.0.6) also supports applying the BLAST compositional matrix adjust scoring procedure^24^ to compute BLAST-like alignment scores (options --comp-based-stats^3,4^).

As an alternative, DIAMOND (v2.0.7) also includes the option to compute full-matrix instead of banded Smith–Waterman extensions (command line option --ext full), which are also vectorized using the SWIPE algorithm.

#### Frameshift alignments

Reads produced by MinION technology^25^ are known to be noisy and contain frequent indel errors, a problem that also translates to assemblies derived from such long reads. In consequence, genes cannot be detected reliably on such DNA sequences. DIAMOND addresses this issue by providing frameshift alignments in translated search (blastx) mode. The protein sequences corresponding to all three reading frames of a strand are aligned simultaneously against the target sequence, allowing shifts in the reading frame at any position in the alignment, while incurring a user-defined score penalty (set using -F on the command line). The raw MinION reads and contigs up to the length of full bacterial chromosomes are supported as input in translated search mode, enabling gene discovery and annotation in the absence of known gene boundaries.

#### Repeat masking

Differentiating between true evolutionary relationships and spurious similarities presents a big challenge in remote homology detection, particularly given the repetitive nature of sequence regions found in many genomes. When dealing with an increasing load of available genomes for tree-of-life scale sequence searches, the ability to differentiate between similarity relationships based on sequence repetitiveness and homology based on a biologically meaningful sequence structure (non-repetitive sequence under purifying selection) becomes crucial to reduce the number of false-positive hits and increase alignment specificity at scale. Masking of low-complexity regions (repeat masking) is the most commonly used strategy to eliminate false-positive hits and to retain only hits found in biologically meaningful homologs. It has been shown that despite using the SegMasker tool included in BLASTP^26^, many more and stronger spurious similarities will arise than are expected on random sequences, as defined by an *e*-value threshold parameter^27^. DIAMOND reduces this false-positive bias by using more stringent and more sophisticated masking paradigms based on tantan. If required, the tantan masking can be replaced by the more conservative default BLASTP SEG masking and composition-based statistics using the option --comp-based-stats 3 (ref. ^24^).

#### Distributed-memory parallelization

As part of DIAMOND, our comprehensive sequence search framework supports a distributed-memory parallelization to leverage the computing power of state-of-the-art HPC and cloud-computing resources for massive-scale protein alignments. To this end, both the query database and the reference database are segmented into data packages that we refer to as chunks. The Cartesian product of both query and reference sets defines a (typically large) set of work packages. In the first instance, files containing metadata on these work packages are created centrally before a parallel run is started on independent computing nodes and are subsequently processed in a distributed manner by multiple worker processes of DIAMOND. Usually, only one worker process runs per compute node, efficiently utilizing all of the locally available cores via threads. Unlike related work such as mpiBLAST^28^, our implementation does not use any special interprocess communication libraries, such as the message passing interface (MPI) specific to HPC environments, instead it relies on input–output operations supported by any POSIX-compliant parallel file system that is mounted on all of the compute nodes involved. The advantage of this approach is that work packages are distributed in a self-organized way at run time to all participating worker processes using simple file-based stacks located in the parallel file system, with atomic push and pop operations. Once all database chunks for a specific query chunk have been processed, the final worker process involved in the query chunk takes on the role of performing the join operation to ultimately create the output stream. Note that the largest part of the temporary files stays local to a compute node, and only the lightweight work-stack files and the DIAMOND hits from the protein searches are written into the shared parallel file system. This strategy significantly reduces input–output overloads and enables massively parallel processing of DIAMOND runs. In addition to the lack of complex dependencies, such as on MPI, we highlight the particular advantages of our approach. First, there is no designated primary worker to induce a bottleneck due to synchronization, or to act as a potential single point of failure. Second, and by design, worker processes may join and leave at run time, which is less important on classical HPC systems that use batch systems to orchestrate potentially large numbers of processes, but is of striking advantage on elastic cloud-computing resources and on existing commodity resources such as networked laboratory desktop computers. Last, our transactional file-based work-distribution protocol enables fault tolerance, which means that if worker processes die unexpectedly, other processes in a subsequent run can take on and resume their work packages.

### Benchmarks

#### Main benchmark

To create a benchmark database, we annotated the 14 September 2019 release of UniRef50 containing 37.5 million sequences with SCOP families. To categorize each protein sequence, we ran SWIPE^22^ using an *e*-value cut-off of 10^−5^ against the SCOPe ASTRAL40 v2.07 dataset^12^ of domain sequences consisting of 4,850 protein families, which resulted in a collection of 7.74 million annotated protein sequences. We used the hit with the highest bit score per SCOPe fold (a grouping of structurally similar superfamilies) to infer the protein family annotation while allowing multidomain associations.

Given that DIAMOND requires a large query dataset to reach its maximum efficiency, we used an analogous SWIPE approach and annotated the NCBI nr database from 25 October 2019 in accordance with SCOPe families. We used UPGMA clustering^29^ on the sets of all protein sequences annotated with the same superfamily to cluster and reduce them to a maximum of 1,000 sequences, which we selected as representatives of that superfamily, resulting in a benchmark dataset of 1.71 million queries.

Both query and reference sequences were locally shuffled in 40-letter windows outside the annotated ranges. All benchmark datasets and annotations have been published^30^.

Alignment for all tools was run on an AMD Ryzen Threadripper 2970WX 24-core workstation clocking at 3.0 GHz with 256 GB of RAM, except for the BLASTP (v2.10.0) run, which, due to its run time limitations on a desktop computer workstation, was performed on the Max Planck Society’s Draco supercomputer at Garching, Germany, using 24 nodes (32 cores on two Intel Haswell E5-2698v3 chips per node). On the benchmark machine the performance of BLASTP (v2.10.0) was estimated using a random subset of 10,000 queries sampled from the initial benchmark dataset.

For each query, we determined the AUC1 value, defined as the number of alignments against sequences matching the query’s protein family, divided by the total number of database sequences of that family (also called the coverage of the protein family). Only hits until the first alignment against a false positive were taken into account, which was defined as the alignment of query and subject sequences from different SCOPe folds. For multidomain proteins, the AUC1 value was averaged over the domains. The AUC1 values of the individual queries were again averaged over the query dataset to obtain the final sensitivity value (Fig. 1a). To ensure that a false positive is contained in the result list of every query, the tools were configured to report all alignments up to an *e*-value of 1,000 (Supplementary Information). Further information about the benchmark design can also be found in the Nature Research Reporting Summary.

#### Detailed assessment of sequence identities in true-positive alignments

We explored the sensitivity of all compared tools in more detail by resolving it at the level of amino acid sequence identity of true-positive alignments. For this purpose, we define the sequence identity of a query–subject association induced by annotation with the same SCOPe protein family as that obtained from the Needleman–Wunsch alignment between the pair of annotated ranges in the query and subject. Extended Data Figure 2 shows a breakdown of the AUC1 sensitivity for our main benchmark, computed as if the search space of positive cases were restricted to associations of the respective sequence identity ranges. Additionally, Extended Data Fig. 3 shows how a query sequence’s family associations are distributed across the identity bins for our benchmark dataset.

#### Supplementary benchmarks

We report benchmark results for two additional datasets, consisting of sequencing reads from Illumina HiSeq 4000 paired end sequencing (2 × 150 base pairs) and Illumina HiSeq 2500 paired end sequencing (2 × 250 base pairs). The datasets were created based on data from a recent rumen metagenome study^31^ (Supplementary Information, see Supplementary Benchmark 1) and an environmental study of the topsoil microbiome^32^ (Supplementary Information, see Supplementary Benchmark 2). SCOPe-annotated datasets of 1.55 million and 1 million reads, respectively, were obtained as described in the Supplementary Information. The benchmark runs for the two query read datasets were carried out analogously to the run for our main benchmark, operating all tools in translated search mode against the same database of SCOPe-annotated UniRef50 sequences. We report performance, AUC1 values and ROC curves for both runs (Extended Data Figs. 4–7).

### Experimental study

The ultimate ambition of DIAMOND v2.0.7 is to provide a comprehensive search framework for sensitive tree-of-life scale protein alignments in the Earth BioGenome Project era and beyond. Although BLAST-like sensitivity levels are the maximally achievable thresholds for pairwise alignments, the next focus of any aligner should be the computational scalability to process millions of sequenced species. With the new --ultra-sensitive mode introduced in DIAMOND v2.0.0 we achieve this critical BLAST-like sensitivity level while maintaining an 80-fold computational speedup, and we achieve an additional near-linear parallel speedup when using the custom DIAMOND HPC implementation. To simulate all facets of a tree-of-life scale protein search that is able to mimic future applications of large-scale comparative genomics projects, we performed DIAMOND --very-sensitive and --ultra-sensitive searches on 520 nodes of the Cobra supercomputer of the Max Planck Society (40 cores on two Intel Skylake 6148 chips, and 192 GB RAM per node), totaling 20,800 computing cores (41,600 threads), using the NCBI nr database (currently storing all sequenced proteins for ~12,000 eukaryotic species and all proteins from ~440,000 genomes of non-eukaryotic species) as the query database, and UniRef50 as the reference dataset. We randomly shuffled the sequences in both FASTA files to avoid a load imbalance due to a biased distribution of sequences in the original files. As a result, DIAMOND v2.0.0 produced 23.1 billion pairwise alignments in the --ultra-sensitive case and 23.0 billion pairwise alignments in the --very-sensitive case, starting from an initial query dataset that contained 281 million sequences and a reference dataset that contained 39 million subject sequences. In --very-sensitive mode the run terminated in 5.42 hours, while in --ultra-sensitive mode it terminated in 17.77 hours. The latter run is shown in Fig. 2 and Extended Data Fig. 1, demonstrating the massive parallelism achieved on the HPC infrastructure, as shown by the processing of individual tasks over time. Due to the parallel nature of the align and join operations, the parallel speedup is virtually linear and is limited only by the throughput of the shared parallel file system of the supercomputer used. This demonstrates that DIAMOND v2.0.0 can harness its algorithmic improvements and its new HPC support to cover all sequenced species in the tree of life within hours rather than months, while matching the alignment sensitivity levels of BLAST. The uncompressed output generated by this run occupies ~1,100 GB of disk space and stores the 100 best protein hits for each sequence in the NCBI nr database.

We envision that in the future this type of DIAMOND output will be easily accessible to all life scientists via a web application in which users can filter and search for their protein homologs of interest within minutes across the tree of life on a precomputed dataset, instead of having to perform complex data analytics and months’ or years’ worth of BLAST searches to obtain sensitive protein alignments at this scale.

### Reporting Summary

Further information on research design is available in the Nature Research Reporting Summary linked to this article.

## Online content

Any methods, additional references, Nature Research reporting summaries, source data, extended data, supplementary information, acknowledgements, peer review information; details of author contributions and competing interests; and statements of data and code availability are available at 10.1038/s41592-021-01101-x.

## Supplementary information

Supplementary Information

Reporting Summary

## Data Availability

The sequence and annotation data that support the findings of this study are available in figshare (10.6084/m9.figshare.c.5053112.v1). The SCOPe ASTRAL40 dataset can be downloaded at http://scop.berkeley.edu/downloads/scopeseq-2.07/astral-scopedom-seqres-gd-sel-gs-bib-40-2.07.fa. The UniRef50 database can be downloaded from ftp://ftp.uniprot.org/pub/databases/uniprot/uniref/uniref50/uniref50.fasta.gz and the NCBI nr database can be downloaded from ftp://ftp.ncbi.nlm.nih.gov/blast/db/FASTA/nr.gz. The sequencing reads of the supplementary benchmarks are part of the samples with European Nucleotide Archive (ENA) accessions SAMEA5383815, SAMEA5383897, SAMEA5383886, SAMEA5383828, SAMEA5383925, SAMEA5383848, SAMEA5383824, SAMEA5383873, SAMEA5384011, SAMEA5383807, SAMEA103892455, SAMEA103892562, SAMEA103892552, SAMEA103892441, SAMEA103892588, SAMEA103892582, SAMEA103892581, SAMEA103892571, SAMEA103892491, SAMEA103892619. Source data are provided with this paper.

## Source data

Source Data Fig. 1 Run time and sensitivity statistics of benchmarking runs, and data points consisting of coverage and errors per query that are the basis of the ROC curves. Source Data Fig. 2 Scaling of DIAMOND on compute clusters. Source Data Extended Data Fig. 2 Underlying data for box plots showing the AUC1 sensitivity resolved by sequence identity used for Extended Data Fig. 2. Source Data Extended Data Fig. 3 Underlying data for box plots showing the family distribution across sequence identity used for Extended Data Fig. 3. Source Data Extended Data Fig. 4 Run time and sensitivity statistics of benchmarking runs used for Extended Data Fig. 4. Source Data Extended Data Fig. 5 Run time and sensitivity statistics of benchmarking runs used for Extended Data Fig. 5. Source Data Extended Data Fig. 6 Data points consisting of coverage and errors per query that are the basis of the ROC curves used for Extended Data Fig. 6. Source Data Extended Data Fig. 7 Data points consisting of coverage and errors per query that are the basis of the ROC curves used for Extended Data Fig. 7.

## Extended data

are available for this paper at 10.1038/s41592-021-01101-x.

## Code availability

The source code of DIAMOND v2.0.7 is available at https://github.com/bbuchfink/diamond and in figshare (10.6084/m9.figshare.14071334.v1).
