# Supplementary material for: Sensitive protein alignments at tree-of-life scale using DIAMOND
Source: Nat Methods. 2021 Apr 7;18(4):366–8. doi: 10.1038/s41592-021-01101-x (PMC8026399; doi:10.1038/s41592-021-01101-x)
Supplement: Supplementary file 1 — Supplementary Information [file 41592_2021_1101_MOESM1_ESM.pdf]

nature portfolio

https://doi.org/10.1038/s41592-021-01101-x

Supplementary information

Sensitive protein alignments at tree-of-life scale using DIAMOND

In the format provided by the authors and unedited

Supplementary Information

Benchmark

Data

The benchmark data is available according to (**Data Availability Statement)**.We provide the two FASTA files that were used as query (query_shuffled.faa.gz) and database (uniref50_annot_shuffled.faa.gz), as well as the query files for the supplementary benchmarks (query_suppl1.fna.gz, query_suppl2.fna.gz). Furthermore, the files query_scop_annotation.tsv.gz, query_suppl1_scop_annotation.tsv.gz, query_suppl2_scop_annotation.tsv.gz and uniref50_scop_annotation.tsv.gz contain our annotation of the query and database sequences with SCOP domains in TSV format. They consist of 7 columns: accession of the SCOP domain, accession of the annotated sequence, SWIPE bit score of annotation, SCOP class, SCOP fold, SCOP superfamily, SCOP family. When downloading the figshare file links using wget, we recommend the option `--content-disposition` to retrieve complete file names.

Evaluating a TSV-formatted alignment output file according to our benchmark may be done using this command line call:

```
diamond roc --family-map uniref50_scop_annotation.tsv.gz --family-map-query
query_scop_annotation.tsv.gz -q <input file>
```

When running this command, the query accessions, along with their individual AUC1 values, will be written to the standard output in tabular-separated format. Note that due to a particularity in formatting accessions, the parameter `--cut-bar` needs to be added to this call when processing MMSeqs2-generated alignment files, or when using makeblastdb with the option `-parse_seqids`. The option `--check-multi-target` needs to be added when processing output files by MMSeqs2 in blastx mode.
The raw data for ROC curves can be generated by adding the option `--roc-file <output file>` to the command line. The lines of the output file will contain tuples of family coverage and error count, which are sums that still need to be divided by the number of queries. When using this option, the third column in the input file is required to be the e-value.

Supplementary benchmarks

For **Supplementary Benchmark 1**, we selected 10 of the metagenome samples at random (sample accessions SAMEA5383815, SAMEA5383897, SAMEA5383886, SAMEA5383828, SAMEA5383925, SAMEA5383848, SAMEA5383824, SAMEA5383873, SAMEA5384011, SAMEA5383807) and randomly sampled 1.5 million 150bp reads from each sample. We annotated these reads by running BLASTX using an e-value cutoff of 10^{-5} against the SCOPe ASTRAL40 v2.07 dataset, resulting in a final query dataset of 1.55 million annotated reads. For **Supplementary Benchmark 2**, we selected 10 of the samples at random (sample accessions SAMEA103892455, SAMEA103892562, SAMEA103892552, SAMEA103892441, SAMEA103892588, SAMEA103892582, SAMEA103892581, SAMEA103892571, SAMEA103892491, SAMEA103892619) and annotated a total of 10.7 million 250bp reads from these samples in the same manner as described above, yielding a query dataset of 1,753,358 annotated reads that we subsampled to 1,000,000 reads.

ROC curves

We created ROC curves that plot the coverage of a protein family against the rate of errors/query depending on the e-value threshold from the alignment output files. Given the result list of a query, we assign each hit to a bin defined by the binary logarithm transformed value of the hit's e-value, rounded to the nearest integer. Alignments with e-value 0.0 are assigned to the lowest bin. Traversing the result list sorted by e-value in ascending order, we determine the coverage of the protein family and the count of false positives that are reached up until a given bin, which we project as a point within the ROC curve for each bin. The tuples of coverage and error count are averaged over all queries for corresponding e-value bins to produce the final ROC curve for the dataset (**Fig**. **1b**).

For the purpose of computing an AUC (Area under the curve), we consider the function that maps the decadic logarithm value of the error rate to the coverage. Values with a true error rate of exactly zero (for which no logarithm can be computed) were discarded since they only correspond to a negligible amount of coverage for our data. The integral of this function is then approximated using the trapezoid rule (**Source Data for Fig. 1**).

We limited the ROC curve and AUC computation at an error rate of 100 errors/query since we do not consider searches at higher error rates of much practical importance.

Detailed assessment of sequence identities in true positive alignments

We define the sequence identity of a query-subject association by Needleman Wunsch alignment between the pair of annotated ranges in the query and subject. This definition is motivated by the fact that since local alignments can be arbitrarily short, the identity of a local alignment would in general not be a meaningful measure. The Needleman Wunsch alignments were computed using the needleall tool from the EMBOSS package (Rice, P., Longden, I. & Bleasby, A. EMBOSS: the European Molecular Biology Open Software Suite. *Trends Genet.* **16**, 276–277, 2000) using the options `-aformat markx10 -gapopen 10.0 -gapextend 0.5 -nobrief -minscore -10.0`.

Environment

The benchmark machine was a AMD Ryzen Threadripper 3960X 24-Core workstation with 256 GB of RAM, a Samsung 860 PRO 4 TB internal SSD, a Seagate FireCuda 520 2 TB M.2 SSD and two Seagate IronWolf Pro 16 TB HDDs in a RAID0 array. The operating system was Ubuntu Linux 20.04.1 LTS. All benchmarked tools were natively compiled from source using GCC 9.3.0.

Programs

BLAST

For all benchmarks and analyses, we used BLAST version 2.10.0 (date 2019/12/04) downloaded from ftp://ftp.ncbi.nlm.nih.gov/blast/executables/blast+/2.10.0/. The command line options were specified as following:

```
blastp -num_threads 48 -evalue 1000 -outfmt 6 -max_target_seqs 100000000
```

For the supplementary benchmarks, the parameters were the same except for replacing blastp by blastx.

QuickBLAST

We downloaded QuickBLAST version 0.0.0 from ftp://ftp.ncbi.nlm.nih.gov/blast/demo/quickblastp.tar.gz. The command line options were specified as following:

```
kblastp -num_threads 48 -evalue 1000 -outfmt 6 -max_target_seqs 100000000
```

When using our complete query file as input, the program was killed due to running out of memory after some time, possibly caused by a memory leak in the code. We worked around this issue by partitioning our query file into 194 smaller FASTA files that we submitted as sequential runs.

MMSeqs2

The benchmark was run with MMSeqs2 commit hash e9678f625b16a806e1ae0bf04d7daf733f1142f2 (date 2020/04/22) cloned from the repository at https://github.com/soedinglab/MMseqs2/. This commit is more recent than release 11 of the tool, which we attempted to use at first, but which showed some dysfunctionalities that appeared to have been fixed in the later commits.

The main parameter for controlling the tool's sensitivity is `-s`, which can be set in a range of 1.0 to 7.5 according to the command line documentation. We chose values of 1.0, 2.5, 6.0 and 7.5 to roughly mimic three of the four sensitivity modes of DIAMOND which are default (fast), `--sensitive`, and `--very-sensitive`.

In MMSeqs2, the `--max-seqs` parameter determining the maximum number of targets for which extensions will be computed for each query also affects the alignment sensitivity and needs to be manually set by the user. Setting this parameter to the number of sequences in the database (which would be the ideal scenario) does not lead to an efficient operation of the program. Hence, we ran the program in its most sensitive mode (`-s 7.5`) with a range of values for this parameter on a subset of 50,000 sequences randomly sampled from our query dataset against our benchmark database in order to determine a suitable value for this parameter. This choice allowed us to obtain the fairest and optimal mode of MMseqs2 operation for sensitivity comparisons with DIAMOND.

`--max-seqs`	AUC1
6250	0.586
12500	0.595
25000	0.599
50000	0.602
100000	0.603
200000	0.604

We concluded that a setting of `--max-seqs 12500` provided a sufficient saturation of the sensitivity but also conducted another run using `--max-seqs 100000 -s 7.5` in order to max out the sensitivity of MMSeqs2 and provide a comparison to the `--ultra-sensitive` mode of DIAMOND.

We carried out analogous computations for supplementary benchmarks 1 and 2 using a subset of 100,000 and 25,000 queries respectively (choice of `--max-seqs` parameter as highlighted):

`--max-seqs`	AUC1 (benchmark 1)	AUC1 (benchmark 2)
2000	0.194	0.195
4000	0.207	0.216
8000	**0.213**	0.230
16000	0.217	0.239
32000	0.220	**0.246**
64000	-	0.254
128000	-	0.258
256000	-	0.258

The benchmark runs can be reproduced using these command line calls:

```
# database creation
mmseqs createdb uniref50_annot_shuffled.faa.gz uniref50_annot_shuffled
mmseqs createdb query_shuffled.faa.gz query_shuffled
# alignment s=2.5
mmseqs search query_shuffled uniref50_annot_shuffled out . --db-load-mode 1 -e 1000
--max-seqs 12500 -s 2.5
# alignment s=6.0
mmseqs search query_shuffled uniref50_annot_shuffled out . --db-load-mode 1 -e 1000
--max-seqs 12500 -s 6.0
# alignment s=7.5
mmseqs search query_shuffled uniref50_annot_shuffled out . --db-load-mode 1 -e 1000
--max-seqs 12500 -s 7.5
# alignment s=7.5 max-seqs=100000
mmseqs search query_shuffled uniref50_annot_shuffled out . --db-load-mode 1 -e 1000
--max-seqs 100000 -s 7.5
# output conversion
mmseqs convertalis query_shuffled uniref50_annot_shuffled out out.tsv --format-
output query,target,evalue
```

Time needed for database file construction and the `convertalis` call to convert the output files into text format was not included in the runtime. The command lines for the supplementary benchmarks were analogous except for setting the `--max-seqs` parameter according to the table above.

DIAMOND (v0.7.12)

DIAMOND v0.7.12 was downloaded from the repository at https://github.com/bbuchfink/diamond/releases/tag/v0.7.12. The command line options were specified as following:

```
# database construction
diamond makedb --in uniref50_annot_shuffled.faa.gz -d uniref50_annot_shuffled -b20
```

```
# alignment in fast mode
diamond blastp -q query_shuffled.faa.gz -d uniref50_annot_shuffled -a out -e 1000 -
k 100000000 -c 1 --log --single-domain
# alignment in sensitive mode
diamond blastp -q query_shuffled.faa.gz -d uniref50_annot_shuffled -a out -e 1000 -
k 100000000 -c 1 --log --single-domain --sensitive
# output conversion
diamond view -a out.daa -o out.tsv
```

Time for the database construction and converting the DAA output files to text format was not included in the runtime.

DIAMOND (v0.4.7)

DIAMOND (v0.4.7) was downloaded at https://static-content.springer.com/esm/art%3A10.1038%2Fnmeth.3176/MediaObjects/41592_2015_BFnmeth3176_MOESM6_ESM.zip. The command line options were specified as following:

```
# database construction
diamond makedb --in uniref50_annot_shuffled.faa -d uniref50_annot_shuffled -t 48
# alignment in fast mode
diamond blastx -q query_suppl1.fna -d uniref50_annot_shuffled -o out.tsv -k
100000000 -t 48 --min-score 25 -r
# alignment in sensitive mode
diamond blastx -q query_suppl1.fna -d uniref50_annot_shuffled -o out.tsv -k
100000000 -t 48 --min-score 25 -r --sensitive
```

DIAMOND (v2.0.7)

DIAMOND version 2 is available from our repository at https://github.com/bbuchfink/diamond. The CMake option `-DCMAKE_BUILD_MARCH=native` was used for native compilation. Note that the CMake option `-DSTRICT_BAND=OFF` was also used, which slightly improves extension performance but also leads to slight deviations of results depending on whether the host system supports SSE4.1, AVX2 or neither. The benchmark runs can be reproduced using these command line options:

```
# database construction
diamond makedb --in uniref50_annot_shuffled.faa.gz -d uniref50_annot_shuffled
# alignment in fast mode
diamond blastp -q query_shuffled.faa.gz -d uniref50_annot_shuffled -o out.tsv -f 6
qseqid sseqid evalue -e1000 -k0 -b20 -c1
# alignment in sensitive mode
diamond blastp -q query_shuffled.faa.gz -d uniref50_annot_shuffled -o out.tsv -f 6
qseqid sseqid evalue -e1000 -k0 -b20 -c1 --sensitive
# alignment in very-sensitive mode
diamond blastp -q query_shuffled.faa.gz -d uniref50_annot_shuffled -o out.tsv -f 6
qseqid sseqid evalue -e1000 -k0 -b0.8 -c1 --very-sensitive --ext banded-fast
# alignment in ultra-sensitive mode
diamond blastp -q query_shuffled.faa.gz -d uniref50_annot_shuffled -o out.tsv -f 6
qseqid sseqid evalue -e1000 -k0 -b0.8 -c1 --ultra-sensitive
```

The options for the supplementary benchmarks were chosen analogously except for calling `diamond blastx` instead of `diamond blastp` and using a larger block size (-b4) for supplementary benchmark 1.

Resource use

We report the maximum resident set size as well as the total disk I/O (data read + written) for the benchmarked tools with respect to our main benchmark.
The tools were adjusted to max out the resources of the benchmark machine, and these numbers should not be seen as minimum requirements. The largest part of DIAMOND's disk I/O consists of reading and writing temporary files, which was handled using a fast local SSD on the benchmark machine. Alternatively, when running the tool in a cluster environment without fast local storage being available, we recommend using a RAM drive for keeping the temporary files (option `-t /dev/shm` on the command line). The block size parameter (option `-b`) can be adjusted to keep the temporary space usage below the necessary limit such that it fits into RAM. This solution will effectively eliminate most of DIAMOND's disk I/O and was also used in our 520 node run (**Experimental Study**).

**Program**	**Mode**	**maxRSS (GB)**	**Read size (GB)**	**Write size (GB)**
DIAMOND v2.0.5	default (fast)	48	14	41
DIAMOND v2.0.5	sensitive	52	98	235
DIAMOND v2.0.5	very-sensitive	55	690	903
DIAMOND v2.0.5	ultra-sensitive	81	4,686	4,656
MMSeqs2	s=1.0	29	11	26
MMSeqs2	s=2.5	37	22	61
MMSeqs2	s=6.0	155	300	430
MMSeqs2	s=7.5	133	303	461
MMSeqs2*	s=7.5	204	2,451	2,727

*using --max-seqs 100000
